# Clinical characteristics and outcome of very elderly patients ≥90 years in intensive care: a retrospective observational study

**DOI:** 10.1186/s13613-015-0097-1

**Published:** 2015-12-21

**Authors:** Sophie Becker, Jakob Müller, Geraldine de Heer, Stephan Braune, Valentin Fuhrmann, Stefan Kluge

**Affiliations:** Department of Intensive Care Medicine, University medical center Hamburg-Eppendorf, Martinistr. 52, 20246 Hamburg, Germany; Department of Anesthesia, University medical center Hamburg-Eppendorf, Hamburg, Germany

**Keywords:** Intensive care, Prognosis, Long- term outcome, Over 90 years old

## Abstract

**Background:**

Since the overall prognosis of very elderly patients is generally limited, admissions to intensive care in these patients are often restricted. Therefore, only very few information is available on the prognosis of nonagenarians after intensive care treatment. The aim of this study was to analyze the clinical characteristics and outcomes of very elderly patients (≥90 years) admitted to an intensive care unit (ICU).

**Methods:**

Monocentric, retrospective observational study of all patients aged ≥90 years admitted to the Department of Intensive Care Medicine with a total capacity of 132 ICU beds at the University Medical Center Hamburg in Germany between January 2008 and June 2013. A multivariate Cox regression analysis was used to identify risk factors for 28-day outcome.

**Results:**

A total of 372 patients ≥90 years of age were admitted to one of the departments ICUs. The majority of patients (66.7 %) were admitted as an emergency admission, of which half underwent unscheduled surgery. 39.8 % of patients required support by mechanical ventilation and vasoactive drugs, and 1.9 % of patients received renal replacement. ICU and hospital mortality rates were 18.3 and 30.9 %, respectively. Overall survival at 1 year after hospital discharge was 34.9 %. Multivariate Cox regression analysis revealed creatinine, bilirubin, age, and necessity of catecholamines as independent risk factors and scheduled surgery as protective factor for 28-day outcome.

**Conclusion:**

Nearly 70 % of patients aged ≥90 years were discharged alive from hospital following treatment at the ICU, and more than half of them were still alive 1 year after their discharge. The results suggest that 1-year survival prognosis of very old ICU patients is not as poor as often perceived and that age per se should not be an exclusion criterion for ICU admission.

Trial registration: WF-0561/13

## Background

As a result of demographic transition, the proportion of elderly and very elderly patients is increasing in industrial countries. Especially the percentage of the oldest patients (>80 years) is growing among the elderly population [1]. In 2030, the worldwide number of nonagenarians (≥90 years) is expected to reach 30 million [2].

Medical progress increasingly allows elderly patients to undergo procedures and operations that only a few decades ago were not feasible because of age [3, 4]. As a result, more very elderly patients are admitted to intensive care units (ICU). However, there is evidence that older patients have a poorer prognosis than younger patients [5–7]. Since the overall prognosis of very elderly patients is generally limited, ICU admissions in these patients are often restricted. Among intensivists and in the literature, the discussion about the appropriateness of ICU admissions of elderly patients is controversial [7–10], due to costs, limited resources, and questionable outcome.

Although international publications indicate that people 80 years of age and older already represent 15 % of all ICU patients [5, 11], there is still a lack of information on prognosis and outcome, especially the older the patient is. Only few studies on elderly patients in intensive care have included nonagenarians, who if included, only accounted for a small proportion of the study population. Especially long-term outcomes have not been studied.

This study investigated, to the best of our knowledge, the largest cohort of nonagenarians treated in the ICU and aims to analyze a large cohort of ≥90-year-old patients and their outcomes and risk factors influencing outcome.

## Methods

### Setting

The University Medical Center Hamburg-Eppendorf is a tertiary-level medical center with 1460 hospital beds and a volume of more than 80,000 in-patients per year. The Department of Intensive Care Medicine includes 11 ICUs with a total capacity of 132 ICU beds. Approximately, 8000 patients are admitted to the department per year, with an average length of stay in the ICU of 4.5 days. The Department of Intensive Care Medicine serves all adult critically ill patients of the university hospital and offers the maximum level of treatment to medical and surgical ICU patients.

### Study design

All patients ≥90 years admitted to our department between 1 January 2008 and 30 June 2013 were eligible for study inclusion. If a patient was admitted to the ICU several times, this was considered as one case, and admission data only for the first ICU admission were analyzed. The following data were extracted from the electronic patient data management system [Intregrated Care Manager© (ICM), Dräger Medical, Lübeck, Germany]: Age, gender, place of residence, the presence of an advance directive, main reason of admission, comorbidities, length of ICU and hospital stay, treatment modalities and organ support (mechanical ventilation, use of catecholamines, renal replacement therapy, blood transfusions, antibiotics), discharge information, ICU- and hospital mortality as well as the occurrence of withholding life support.

Severity of illness was assessed using the Simplified Acute Physiology Score II (SAPS II). ICU and hospital mortality were analyzed, and the main outcome variable was 28-day mortality. ICU mortality rates were compared to those of all patients between 80 and 89 years admitted to the ICU during the study period.

To obtain survival data at 1 year after hospital discharge, we contacted survivors or their relatives by phone. If the patient or the next of kin could not be contacted, information was obtained from the patients’ general practitioner, their nursing homes, or the registration office.

The study was approved by the institutional review board (ethics committee of the Hamburg Chamber of Physicians, WF-0561/13). Due to the retrospective character of the study, patient’s consent was not necessary according to local requirements.

### Statistics

Data are presented either as median and interquartile ranges (IQR) or as absolute numbers with percentages. Binary variables were compared with Chi Square-Analysis or Fisher’s exact, as appropriate. Metric variables were compared with the Mann–Whitney-U-Test. 28-day survival was assessed using the Kaplan–Meier method and Cox proportional hazard regression model. The Kaplan–Meier method was used to estimate survival curves, and log-rank test was used to test for differences between survival curves. The results of the Cox proportional hazard regression analysis are expressed with hazard ratios (HR). We included following parameters in the analysis: sex, scheduled surgery, unscheduled surgery, medical admission, mechanical ventilation, catecholamine therapy, renal replacement therapy, age, pH, leukocytes, creatinine, bilirubin, and hemoglobin. Parameters that were significant in prediction for 28-day mortality in the univariate analysis (p < 0.05) were included in the multivariate analysis. A two-sided *p* value of <0.05 was considered statistically significant. Statistical analysis was conducted using IBM SPSS Statistics Version 20.0.

## Results

A total of 34,392 patients were treated in the Department of Intensive Care Medicine during the study period. A total of 372 (1.1 %) patients were ≥90 years old. The median age was 92.2 years (IQR 91.0–94.2), and the proportion of female patients was 66.7 %. Before ICU admission, 230 patients (61.8 %) lived at home, 128 (34.4 %) in nursing homes, and 14 (3.7 %) at assisted living facilities.

248 patients (66.7 %) were admitted to the ICU as an emergency admission, of which 50 % underwent unscheduled surgery. 33.3 % of patients (n = 124) were admitted following elective surgery. Trauma (28.8 %), cardiac diseases (21.5 %), and gastrointestinal diseases (10.5 %) were the most frequent causes of ICU admission. A detailed list of all patients’ characteristics is shown in Table 1. The average SAPS II score within 24 h of ICU admission was 36 (IQR 29–48). 90.9 % of patients were anemic (Hb < 13 g/dl for men, <12 g/dl for woman), 51.5 % of patients presented with leucocytosis (>11.5 Mrd/l), and 46.7 and 22.9 % showed elevated levels of serum-creatinine and -bilirubin (>1.1 mg/dl), respectively. Acidosis occurred in 53.2 % of cases within 24 h after ICU admission.

**Table 1:** Patient characteristics

Characteristics	All patients	ICU survivors	ICU-non-survivors	p value
Number	372	304	68	
Age (years), MD (IQR)	92.2 (91–94.3)	92.2 (90.9–94.4)	92.3 (91–93.3)	0.858
Female, n (%)	248 (66.7)	211 (69.4)	37 (54.4)	0.018
Unplanned surgery, n (%)	121 (32.5)	101 (33.2)	20 (29.4)	0.544
Planned surgery, n (%)	124 (33.3)	117 (38.5)	7 (10.3)	<0.001
Medical, n (%)	127 (34.1)	86 (28.3)	41 (60.3)	<0.001
SAPS II, MD (IQR)	36 (29–48)	34 (28–43)	55 (44.8–65.8)	<0.001
Admission source, n (%)				
Normal ward	227 (61.0)	195 (64.1)	32 (47.1)	0.009
Emergency room	127 (34.1)	97 (31.9)	30 (44.1)	0.055
Other hospital	18 (4.8)	12 (3.9)	6 (8.8)	0.09
Admission diagnosis, n (%)				
Trauma	107 (28.8)	100 (32.9)	7 (10.3)	<0.001
Cardiac surgery	43 (11.6)	39 (12.8)	4 (5.9)	0.105
Abdominal surgery	39 (10.5)	31 (10.2)	8 (11.8)	0.703
CPR	26 (7.0)	13 (4.3)	13 (19.1)	<0.001
Sepsis	30 (8.1)	19 (6.3)	11 (16.2)	0.007
Pneumonia	11 (3.0)	7 (2.3)	4 (5.9)	0.115
Myocardial infarction	18 (4.8)	13 (4.3)	5 (7.4)	0.285
Arrhythmia and heart failure	19 (5.1)	15 (4.9)	4 (5.9)	0.748
Neurologic	22 (5.9)	15 (4.9)	7 (10.3)	0.09
Cerebral hemorrhage	6 (1.6)	2 (0.7)	4 (5.9)	0.012
Pulmonary embolism	5 (1.3)	5 (1.6)	0	0.589
Surgical, miscellaneous^a^	41 (11.0)	38 (12.5)	3 (4.4)	0.054
Others (medical)^b^	21 (5.6)	16 (5.3)	5 (7.4)	0.582
Comorbidity, n (%)				
Arterial hypertension	246 (66.1)	204 (67.1)	42 (61.8)	0.4
Cardiac diseases^c^	185 (49.7)	150 (49.3)	35 (51.5)	0.751
Chronic heart failure	82 (22.0)	65 (21.4)	17 (25.0)	0.515
Cardiac arrhythmia	116 (31.2)	95 (31.3)	21 (30.9)	0.953
Valvular heart diseases	38 (10.2)	31 (10.2)	7 (10.3)	0.981
Coronary heart disease	95 (25.5)	75 (24.7)	20 (29.4)	0.418
Chronic renal insufficiency	83 (22.3)	64 (21.1)	19 (27.9)	0.217
Neurodegenerative disease	79 (21.2)	68 (22.4)	11 (16.2)	0.259
Diabetes	47 (12.6)	40 (13.2)	7 (10.3)	0.521
Respiratory diseases	45 (12.1)	32 (10.5)	13 (19.1)	0.079
Cancer	28 (7.5)	23 (7.6)	5 (7.4)	0.952
Skeletal system disorders	39 (10.5)	31 (10.2)	8 (11.8)	0.703
Thyroid disorders	39 (10.5)	35 (11.5)	4 (5.9)	0.171
Mental disorders	18 (4.8)	15 (4.9)	3 (4.4)	0.856

*ICU* intensive care unit, *MD* median, *IQR* interquartile range, *CPR* Cardiopulmonary resuscitation

^a^surgical interventions on the spine, blood vessels, skin, and the head and neck region

^b^Renal failure, side-effects of medication, electrolyte imbalance, vascular diseases and diseases of the head and neck region, conservatively treated gastrointestinal bleeding

^c^Cases of >1 heart diseases were regarded as n = 1

### Clinical course

The median length of stay in the ICU and in the hospital was 1.4 days (IQR 0.8–2.7) and 11 days (IQR 7–17), respectively. A total of 148 patients (39.8 %) were mechanically ventilated, of these 34 (9.1 %) were on non-invasive ventilation. Catecholamine support was applied in 148 patients (39.8 %). 25 patients (6.7 %) developed acute renal failure and of these 7 patients (1.9 %) received renal replacement therapy during their ICU stay (Table 2). Five of these patients had been on intermittent hemodialysis for end-stage renal disease already prior to hospital admission. 114 patients (30.6 %) required antibiotic therapy.

**Table 2:** procedures on ICU

Procedure, n (%)	All patients	length (h) MD (IQR)	ICU survivors	ICU-non-survivors	p value
Mechanical ventilation	148 (39.8)	12.5 (4.5–34)	91 (29.9)	57 (83.8)	<0.001
Catecholamines	148 (39.8)	20 (7–43.75)	96 (31.6)	52 (76.5)	<0.001
Blood transfusion	76 (20.4)		57 (18.8)	19 (27.9)	0.089
Renal replacement	7 (1.9)		3 (1)	4 (5.9)	0.007
Total	213 (57.3)		150 (49.3)	63 (92.6)	<0.001

*ICU* intensive care unit, *MD* median, *IQR* interquartile range

### Short-term outcomes

ICU and hospital mortality were 18.3 and 30.9 %, respectively.

**Fig. 1:**
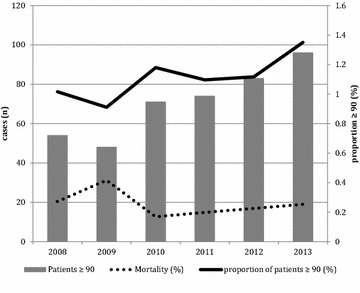
Trends in admission and mortality. Absolute number, mortality, and proportion of nonagenarians in intensive care over time

Non-survivors had higher severity of disease as illustrated by SAPS II (55.8 vs. 36.1), were more likely to be male (25 vs. 14.9 %), and had more frequent unscheduled surgery or medical reasons for ICU admission (Table 1). Annual mortality rates are presented in Fig. 1.

In-patients who were admitted twice (n = 28), hospital mortality was 45.5 %. Five patients (1.3 %) were admitted three times and had a mortality rate of 80 %. 65 patients (17.5 %) had an advance directive. The decision to withhold or withdraw therapy was made in 92 patients (24.7 %). The main area of withholding therapy was pre-existing or subsequently made “Do Not Resuscitate” (73.8 %) and “Do Not Intubate” (47.8 %) orders.

In a Cox regression proportional hazard analysis in regard to 28-day survival creatinine, bilirubin, age, and necessity of catecholamine therapy were independent risk factors for worse 28-day outcome; scheduled surgery was a protective factor. Details are illustrated in Table 3a and 3b.

**Table 3:** Cox regression proportional hazard analysis for factors influencing 28-day survival

Variables	Hazard-ratio (95 % CI)	p value
Univariate analysis for 28-day outcome		
Planned surgery	0.242 (0.143–0.409)	<0.001
Medical admission	2.156 (1.515–3.068)	<0.001
Unplanned surgery	1.357 (0.945–1.948)	0.098
Mechanical ventilation	3.186 (2.216–4.58)	<0.001
Catecholamines	2.602 (1.819–3.722)	<0.001
Renal replacement	1.379 (0.438–4.335)	0.583
Age	1.085 (1.022–1.151)	0.008
Sex (female)	0.753 (0.524–1.081)	0.124
pH	0.829 (0.626–1.097)	0.189
Leukocytes	1.032 (1.007–1.0579	0.01
Creatinine	1.328 (1.168–1.511)	<0.001
Hemoglobin	0.97 (0.872–1.079)	0.576
Bilirubin	1.435 (1.186–1.736)	<0.001
Multivariate analysis for 28-day outcome		
Planned surgery	0.439 (0.225–0.856)	0.016
Medical	1.125 (0.665–1.902)	0.661
Mechanical ventilation	1.513 (0.819–2.796)	0.186
Catecholamines	2.224 (1.195–4.139)	0.012
Age	1.14 (1.045–1.243)	0.003
Leukocytes	1.017 (0.986–1.049)	0.276
Creatinine	1.224 (1.033–1.45)	0.02
Bilirubin	1.281 (1.046–1.569)	0.017

*CI* Confidence interval

All parameters at ICU admission

147 patients (55.1 %) were transferred to other hospitals either for further treatment or rehabilitation before moving to their final discharge destination. Details of outcomes and discharge destinations are shown in Table 4.

**Table 4:** Clinical course and outcome

Results	n	%
ICU mortality^a^	68	18.3
Hospital mortality^a^	115	30.9
Unplanned surgery	46	38
Planned surgery	15	12.1
Medical admission	54	42.5
Withholding and/or withdrawal of therapy	93	25
Discharge destination		
Home	122	47.5
Nursing care facilities	101	39.3
Short-term nursing care	17	6.6
Unknown	17	6.6
28-day mortality	149	40.1
90-day mortality	176	47.3
1-year mortality	242	65.1

^a^The latest stay was considered for calculation

*ICU* intensive care unit

### ICU-outcome in the 80–89 age group

2234 octogenarians were admitted to the ICU in the same period, which account for 6.5 % of all ICU admissions. The median age was 85.6 years (IQR 83.9–87.4), 1288 (57.7 %) were female. Median length of stay was 1.8 days (IQR 0.9–4.0), ICU mortality was 16.6 % (n = 370). ICU mortality did not differ significantly between nonagenarians and octogenarians (p = 0.412).

### Mid- and long-term outcomes

**Fig. 2:**
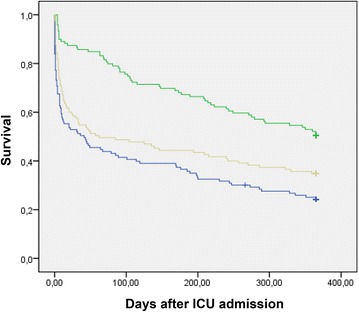
Survival from Intensive care unit (ICU) admission. Kaplan–Meier survival curve: patients after medical admission (*blue*), planned surgery (*green*) and unplanned surgery (*gray*). Groups were compared via Log-rank test (p < 0.01)

Long-term survival follow-up (Fig. 2) was available for 242 of 257 hospital survivors (94.2 %) and overall 357 patients. 3 months and 1 year after discharge, 196 patients (52.7 %) and 130 patients (34.9 %) of the initial study population were still alive. Surgical patients had a better one-year survival than patients admitted for medical reasons (planned surgery: 48.4 %, unplanned surgery: 33.1 % vs. 23.6 % (medical), p < 0.001).

The impact of selected factors in regard to 1 year survival is illustrated in Table 5.

**Table 5:** Factors influencing long-term survival

Parameter	1 year survivors	1-year non-survivors	p value
Age (years), median (IQR)	92.9 (92–95.4)	91.4 (90.7–93.1)	0.101
Female (%)	75.4	61.2	0.006
Unplanned surgery (%)	30.8	33	0.659
Planned surgery (%)	46.2	26	<0.001
Medical (%)	23.1	41	0.001
Admission diagnosis			
Trauma (%)	33.8	25.6	0.095
Gastrointestinal (%)	12.3	8.4	0.229
Tumor (%)	10.0	9.7	0.925
Pulmonary (%)	3.8	7.5	0.168
Neurological (%)	3.8	7.9	0.13
Cardiopulmonary resuscitation (%)	3.1	9.3	0.028
Cardiac (%)	24.6	18.1	0.14
Sepsis (%)	1.5	9.7	0.003
Others	6.9	4	0.219
Comorbidity			
Arterial hypertension (%)	70.0	63.9	0.24
Cardiac diseases (%)	47.7	50.7	0.589
Chronic heart failure (%)	19.2	22.9	0.416
Cardiac arrhythmia (%)	27.7	33.9	0.223
Valvular heart disease (%)	12.3	8.4	0.229
Chronic renal insufficiency (%)	14.6	25.6	0.016
Neurodegenerative diseases (%)	21.8	78.2	0.002
Diabetes (%)	13.1	12.8	0.935
Respiratory diseases (%)	7.7	14.5	0.056
Cancer (%)	5.4	8.8	0.239
Skeletal system disorders (%)	9.2	11	0.595
Thyroid disorders (%)	16.2	7.5	0.011
Mental disorders (%)	1.5	7	0.022
Procedures on ICU			
Mechanical ventilation (%)	26.2	47.6	<0.001
Catecholamines (%)	29.2	45.8	0.002
Blood transfusion (%)	14.6	24.2	0.031
Renal replacement therapy (%)	0.8	2.6	0.219
SAPS 2, median (IQR)	33 (28–44)	42 (33–53)	<0.001
pH, median (IQR)	7.35 (7.29–7.45)	7.34 (7.25–7.45)	<0.001
Bilirubin, median (IQR)	0.7 (0.5–1.1)	0.8 (0.5–1.1)	0.406
Hemoglobin, median (IQR)	9.8 (8.6–10.8)	9.6 (8.2–10.7)	0.016
Leukocytes, median (IQR)	11.9 (8.4–15.7)	11.3 (8.4–15.5)	0.048
Creatinine, median (IQR)	1.1 (0.8–1.4)	1.2 (0.9–1.8)	<0.001

## Discussion

This study evaluated the characteristics and outcomes of the largest cohort of nonagenarians in ICU published to date and provided data on their long-term survival.

Despite the fact that elderly patients are increasingly been treated in the intensive care environment, there is a lack of information available about their prognosis and outcome.

The 81.7 % ICU-, 70 % hospital-, and 35 % one-year survival rates stand in contrast to and challenge widespread beliefs about the poor short- and long-term prognosis of nonagenarians admitted to the ICU. Especially the hospital mortality rates of patients undergoing planned surgery were remarkably low, whereas the outcome worsened after unplanned ICU admission and especially after ICU readmission. Within the group of nonagenarians, creatinine, bilirubin, age, and necessity of catecholamine therapy cause of admission were independent factors for 28-day outcome. Not surprisingly, the 18.3 % ICU mortality of the study group of very elderly ICU patients was higher than that of the departments overall ICU mortality of 9 %. This age-related mortality risk is in line with many other outcome studies on a wide variety of critically ill ICU populations. Whereas several studies have identified age as an independent risk factor for ICU mortality [6, 7, 12–16], other studies have found the severity of illness and comorbidities to be more important risk factors than age itself [5, 17–20].

Analyzing data from a large Austrian database (n = 17,126), Ihra et al. found a significantly higher hospital mortality rate in patients older than 80 years in comparison to patients younger than 80 years (31.0 vs. 15.9 %) [5]. Only few observational studies have analyzed the outcome of the nonagenarians (≥90 years). Demoule et al. examined 36 patients ≥90 years in a French ICU. ICU and hospital mortality were 28 and 47 %, respectively [21]. Rellos et al. analyzed 60 patients ≥90 years in a Greek ICU, which accounted for 1.1 % of all ICU admissions. The average length of stay in ICU and hospital was 5 and 23 days, respectively, with an ICU mortality of 20 % [22]. Other studies with data of patients >85 years demonstrated ICU mortality rates ranging from 14.6 [6] to 36.6 % [7].

The comparability between all these studies is limited by differences in the study settings and health care systems resulting in different ICU admission policies and practices. Additionally, some studies analyzed predominantly elderly patients with unplanned ICU admissions [6], explaining differences in mortality rates between studies. In contrast, the present study included all very elderly patients treated in the ICU. One possible contributing reason for a higher mortality rate in elderly ICU patients is the fact that the decision to limit or withhold therapy occurs more frequently among elderly ICU patients. Accordingly, Seder et al. found increasing rates of withholding and withdrawal of life support in the ICU with advanced age [23], and Al-Dorzi et al. observed a more frequent application of Do Not Resuscitate- orders in patients >80 years [24]. In line with these previous findings, we recorded a quarter of very elderly ICU patients not receiving maximal therapy on the basis of an advanced directive and/or a presumed poor prognosis.

Patients admitted to the ICU following scheduled surgery had lower mortality rates than patients with unscheduled admission. Correspondingly, other studies observed the best outcome in the scheduled surgery group among very elderly patients [22, 25]. Additionally, admission for unplanned surgery was a predictor for poor outcome [25]. The differences in mortality between the three subgroups can be partly explained by the severity of acute illness. Accordingly, we observed the highest mortality rates in patients following medical admission.

At present, the average life expectancy of a 90-year-old German person is 3.8 years for men and 4.3 years for woman, and life expectancy at an age of 95 years still is 2.7 to 3 years [26]. Approximately, one-third of our entire study population was still alive at 1 year after ICU discharge. Similar findings were made by recent studies with one-year survival rates among elderly ICU patients ranging from 28 to 56 % [25, 27–30].

Limited ICU resources are one of the main reasons for controversial discussions about the accessibility of intensive care treatment for elderly patients [9]. However, findings of the recently published ELDICUS study suggest that of all patients, elderly subjects have a high benefit from ICU treatment [12].

Our study cohort represented only 1.1 % of all departmental ICU admissions from 2008 to 2013. However, the proportion of elderly patients is expected to constantly rise as a result of the demographic transition and this will also affect intensive care medicine [5, 11]. Thus, intensivists will increasingly have to cope with the special challenges of an increasingly aging ICU population and related aspects, such as multimorbidity, polypharmacy, and ethical questions. Our patients were hospitalized mainly for traumatic causes and cardiovascular diseases. Corresponding findings were made by prior studies [5–7, 11], especially the incidence of cardiovascular diseases particularly increases with advanced age [31].

The results of our study have to be interpreted with caution due to the following limitations: Because of the single-center study design, results may not be generalizable to other settings. The relatively good survival rates of our nonagenarian ICU patients may have been the result of a preselection bias of restrictions to ICU admission decisions in this age group. This important aspect was outside the scope of this study. Furthermore, our follow-up data do not provide insights into quality of life and functional status after hospital discharge. Other study groups found, that both, quality of life and autonomy in activities of daily living among elderly ICU survivors were deemed to be satisfactory [28, 32]. Further and larger multicenter studies on the long-term outcome of elderly ICU patients with regard to survival and quality of life are warranted.

## Conclusion

Nearly 70 % of patients aged ≥90 years were discharged alive from hospital following treatment at the ICU and more than one-third were still alive 1 year after their discharge. The results suggest that long-term survival prognosis of very elderly ICU patients may be not as poor as often perceived. Chronological age per se should not be an exclusion criterion for ICU admission. Instead, the biological age, an achievable therapeutic goal and the patient’s will ought to play a major role in the decision-making process. Then, intensive care treatment may be justified even for patients with shorter life expectancy than the general population.

## Abbreviations

ICU: intensive care unit
IQR: interquartile range
CI: confidence interval
ROC: receiver operating characteristic
SAPS II: Simplified Acute Physiology Score II
SPSS: Statistical Package for the Social Sciences
